# Machine Learning-Based Classification of Mango Pulp Weevil Activity Utilizing an Acoustic Sensor

**DOI:** 10.3390/mi14111979

**Published:** 2023-10-25

**Authors:** Ivane Ann P. Banlawe, Jennifer C. dela Cruz

**Affiliations:** 1 College of Engineering and Technology, Western Philippines University, Aborlan 5302, Philippines; 2 School of Graduate Studies, Mapua University, Intramuros, Manila 1002, Philippines; jcdelacruz@mapua.edu.ph

**Keywords:** mango pulp weevil, acoustic detection, MEMS, SVM

## Abstract

The mango pulp weevil (MPW) is an aggressive pest that mates seasonally according to the cycle of the mango fruit. After discovering the existence of the mango pulp weevil in Palawan, the island has been under quarantine for exporting mangoes. Detection of the pest proves difficult as the pest does not leave a physical sign that the mango has been damaged. Infested mangoes are wasted as they cannot be sold due to damage. This study serves as a base study for non-invasive mango pulp weevil detection using MATLAB machine learning and audio feature extraction tools. Acoustic sensors were evaluated for best-fit use in the study. The rationale for selecting the acoustic sensors includes local availability and accessibility. Among the three sensors tested, the MEMS sensor had the best result. The data for acoustic frequency are acquired using the selected sensor, which is placed inside a soundproof chamber to minimize the noise and isolate the sound produced by each activity. The identified activity of the adult mango pulp weevil includes walking, resting, and mating. The Mel-frequency cepstral coefficient (MFCC) was used for feature extraction of the recorded audio and training of the SVM classifier. The study achieved 89.81% overall accuracy in characterizing mango pulp weevil activity.

## 1. Introduction

Machine learning has long been applied in agriculture [1], and future technological developments in this industry heavily rely on automation. Applications of machine learning in agriculture include mapping [2,3], crop management [4,5,6], precision irrigation and spraying [7,8,9], livestock management [10,11,12], automated farm management [13,14,15], and pest control [16,17,18,19,20,21]. Machine learning applied to pest control is seen as a long-term solution to different problems in agriculture, such as non-destructive testing of the presence of insects inside the fruit [22,23]. This application was exploited in the study to solve the problem of quarantine pests, especially in Palawan, Philippines.

The Philippines, as the sixth-largest producer of mango in the world, has exported locally grown mangoes worth millions of dollars. Palawan has vast mango plantations but does not contribute to the mango exportation income of the Philippines due to a particular pest, *Sternochetus frigidus* (Fabricius) or the mango pulp weevil (MPW), which was discovered in Southern Palawan in 1987 [24]. Because of this pest, Palawan has been placed under BPI Special Quarantine Order No. 20, which prohibits exporting and shipping mangoes to other parts of the Philippines. After this quarantine was implemented, farmers abandoned their mango plantations, shifting to another crop and furthering the infestation of the pest. The infestation of the MPW in the province rose from 40% to 71% in 2016 and 2017, respectively, in the absence of a wide market. The impact of this infestation translated to a decline in mango production by 30% and a decrease of more than 50% of the yield of one mango tree, amounting to a loss of PHP 2000 per tree [25]. The MPW propagates easily because the female lays the eggs a few minutes after mating and can mate 165 times in their lifetime. The number of eggs laid after mating can be as many as 800 [26,27]. The adult MPW mainly preys on developing fruits by mating on the peel of mangoes and laying their eggs on the surface to grow. At the end of the fruiting period, the grown eggs have already infested the mango internally, showing no outward signs of infestation. Detection is challenging since the fruit needs to be cut open to verify infestation [23,27]. MPW detection has been a long-term problem in Palawan and needs a solution [28]. Current non-destructive measures, such as detection by soft X-rays and irradiation, were the most effective postharvest measures, but detection in earlier stages has not been covered in these previous researches. [23,25,27,29,30,31].

An Integrated pest management (IPM) approach has been formulated specifically for the farmers of Palawan to increase the yield so that mango production in Palawan will continue. IPM consists of cultural methods, such as open-center pruning, sanitary pruning, field sanitation, brush weeding, and chemical methods, such as insecticide application at a reduced level, following the IPM schedule. This method has significantly decreased the infestation of the MPW from 40% to 0% and increased the mango yield, with a net profit of PHP 1890 per mango tree. The study also revealed that a 10-min exposure to sunlight during 10 a.m., 12 p.m., and 2 p.m. is fatal to the MPW [30,32,33]. This IPM was modified in 2013 by [31], aiming to disrupt the habitat of the MPW. This IPM was compared to the farmer’s practice and produced desirable results: the abundance of the MPW was 0%, the fruit fly was reduced to 1.6%, anthracnose was 3%, and stem end rot was 2.9%. The mango yield increased to 175 kg per tree. The IPM method, although effective, is labor-intensive, demands more workforce, and is costly.

Extraction and identification of volatiles were also studied for possible attraction traps. Studies examining the chemical components of the mango flower and fruit that could attract the MPW were conducted by [34,35]. It was found that the MPW responds to a single compound, and the highest response was to acetic acid (66.7%), depending upon the dose. The MPW also responds to a blend of odors consisting of acetic acid (0.7%), decane (0.3%), acetone (4.4%), linalool (82.2%), ethyl benzoate (11.4%), and 2-methyl heptanone (1.0%), which caused a 70% rate of attraction from the MPW [16]. It is recommended that the best timing period is before the fruit reaches 4 cm, which occurs 3–4 weeks after fruiting [17,35,36,37]. Ref. [38] studied the male MPW’s attraction to frass volatiles, and the attraction (73%) was more significant than to floral volatiles (70%). The majority of frass is hexane 2,5 dimethyl (22.90%) and acetic acid (17.77%). Although this could serve as an attractant for the MPW, the chemicals quickly evaporate at higher temperatures.

A mass-rearing technique and mode of transport have been formulated to produce more MPWs for laboratory testing and observations [39]. For mass rearing to be successful, the behavioral characteristics of the MPW were observed, including life span, preferences, and survival conditions. The results showed that MPW can live approximately 16 days without food, bark, or water. But, since MPW are monophagous species, they can only complete their development with mangoes and not other crops [35,40,41]. The same study was performed in India using the *S. gravis* species of the MPW, a timeline of the developmental stages was made, which is as follows: 7–10 days incubation period, 8–10 days first instar larva, 8–9 days second instar larva, 7–8 days third instar larva, 8–9 days fourth instar larva, 8–11 days fifth instar larva, 2 days pre-pupa, 10 days pupa, and 58–60 days adult [27]. The MPW was also observed for 24 h during the off-season stage of the mango to determine their activity. Most of the time, the MPW rests with few other activities, such as crawling a short distance (1–24 inches); even during the rainy season, MPWs rest. Additionally, *Sternochetus* spp. could apparently survive in a climate appropriate for mangoes [26,35,42].

Gamma irradiation was explored after the successful mass-rearing. This was performed to determine the effects of the radiation on MPW, to observe the most tolerant stage of the pest, and to identify the effective dose of radiation to disinfect the mango from MPW. Results showed that irradiation produced sterility in male MPW, the adult is the most tolerant stage, and that 165 Gy is the minimum dose required for the disinfection. It was suggested that irradiation technology be adopted to eliminate MPW in Palawan, but there is still no progress in the technology [43,44,45].

Studying the biology and distribution of an MPW is a prerequisite to synthesizing a solution to the problem. Characterizing the mating activity frequency will provide a better understanding of the MPWs, which could lead to developing pest control [46].

Acoustic technology has long existed in insect characterization and communications studies. These include records of sound produced by insect activities and behavioral communications such as in distress, predation, mating, and by their community. Studies proved that an increase in population density increases acoustic emissions. These data were used to characterize the insects and detect their activity [47,48,49,50].

Insect detection was mainly performed for the stored grain products. One developed system that used Hilbert transform in an audio recording resulted in 100% precision for non-infestation but performed average for infestation detection [48]. A device was developed to detect *S. maize* activity utilizing a microphone, a signal-conditioning circuit, and a microcontroller, resulting in 93% accuracy for the infected maize [51]. Sensors were also explored in detecting the *S. oryzae*, the ceramic vibrational sensor, and the laser Doppler vibrometer. Both sensors clearly detected insects of sizes from 1 mm in a soundproofed box, and the laser vibrometer was sensitive enough to detect larval activity [52].

Automated detection and identification were also explored in this area. A computer-assisted monitoring setup was performed in wheat bins utilizing 140 sensors by [53] to identify the insect pest and estimate the level of infestation. Seven (7) species were found, and the most dominant was the *R. dominica* (82.7%). The insect density can be estimated to be 8.8–11.5 insects per kilogram of wheat. Insects were mainly located in the top center of the bins [53]. An acoustic probe with piezoelectric sensors and an algorithm for characterizing insect activity in grain was developed. It was revealed that the prominent peak of energy frequency for an adult rice weevil ranges from 1.8 to 3.0 kHz and from 1.3 up to 2.0 kHz for the larval stage. Also, the maximum detection distance was determined to be 20 cm for the adult and 15 cm for the larva [54]. A recent study that utilized MFCC and ANN in MATLAB to detect adult rice weevil with 9850 data sets used 70% for algorithm training and 15% for testing and validation, resulting in 94.70% accuracy [55].

Studies have also been performed to detect destructive beetles because their infestation leads to the death of their host tree. These beetles feed on the trunk near the roots, which damages the tree and eventually kills it. More of the activities of the insect in the trunk involve feeding and boring. An adult *Oryctes rhinoceros* was studied in the palm trees of Guam. Accelerometers were attached to 30 cm spikes placed inside the tree trunks, and signals were processed using the DAVIS software. It was found that the signals produced by the adult were impulses with peaks below approximately 2 kHz, and the larvae had the same signal durations, only lower in amplitude [56]. Early detection of the red palm weevil was developed by [57] using an acoustic microphone with −44d sensitivity, and the filtered signals were processed using a PIC16F88 controller. The device is powered by a rechargeable battery to ensure continuous operation for 5 days. The study showed that the spectra of infested palms differ from the non-infested ones. Infested palms have a higher intensity of around 2250 Hz and the intensity of the signal at the specified frequency increases with time. Infested palms produced a signal with voltages higher than 0.7 v [57]. Another detection approach was fixing the recorder to the trunk, and signals were fed into MATLAB utilizing MFCC in feature extraction. This study differentiated the sound of the red palm weevil (RPW) and rhinoceros beetle (RB), with the RPW appearing as sharp clicks and shorter than RB, which have deeper and thicker clicks with shorter lengths. The signals created by the adult RPW were: Min: 184 Hz–33.2 dB Max: 4591 Hz–21.3 dB, while for the RB were: Min: 35 Hz–10.2 dB Max: 42 Hz–3.5 dB [58]. The laser Doppler vibrometer was proven effective in detecting insect movement in tree barks [52].

Detection of pests in soil was explored using an accelerometer attached (5 cm above the ground) to the long spikes buried in the soil under the canopy of the orange tree. The signals were stored in a digital audio tape and processed using custom-written software. The system detected the citrus root weevil feeding sound, as well as movement in several trees, and background levels were at 65 dB [59]. Another study explored the possibility of detection in plant stems using alligator clips to clamp the accelerometer in the plant stem with a counterbalance to keep the stem from bending. This study compared sounds produced by *C. cinctus* in wheat stems with sounds produced by *Metamasius callizona* larvae hidden inside the meristematic tissue of bromeliad. Larvae of both species produce brief, >1 kHz sound, loud enough to be detected by an acoustic sensor in moderate background noise without using an insulated room or anechoic chamber. The improved clamp system is useful in detecting the activity of insects that are hiding inside plant tissue [60]. A system using vibrational sensors was developed and tested for a soundproofed vegetable box, and Kaphra beetle activities were detected [52]. Noise minimization and sound transmission employed in acoustic sensors were studied by [61,62].

Based on the previous studies, an acoustic sensing technique could be used to characterize and detect the MPW since it is a non-destructive procedure used to monitor and sense the movement of the subject. Recent technological developments in acoustic sensing tools included increased accuracy, cost-effectiveness, and adaptability to different field applications, making the method reliable [63].

This study explores the possibility of using locally available acoustic sensors, coupled with recent technology developments, in detecting the presence and classifying the activity of the mango pulp weevil. This study also presents the performance of the SVM algorithm in classifying MPW activity based on emitted frequencies. Characterization of frequencies of MPW for the different activities and the development of a suitable machine learning algorithm for the non-destructive detection of the said pest will lead to reduced labor costs for farmers and minimization of chemical interventions that are known to be hazardous for farmers. The result of this study serves as a baseline for the development of a protocol or a technology to eradicate the pest.

## 2. Materials and Methods

The study used locally available materials in implementing the tests, including the sensors and monitoring equipment, for easy replacement and continuation of the study whenever trouble may be encountered.

### 2.1. A Controlled Environment

**Figure 1:**
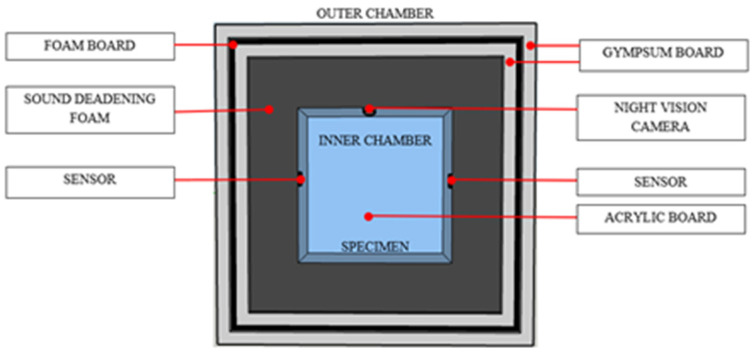
Soundproof chamber design [28].

Most insect detection tests were performed in a laboratory using soundproofing materials [64,65,66]. An isolated soundproof chamber, shown in Figure 1, was developed to study the initial impressions of the mango pulp weevil for each activity. The chamber where the weevils were tested needs to be isolated from the outside to lessen or prevent any gathering of unnecessary noise. The main acrylic box was enclosed on a gypsum board box measuring about 50 cm on each side. Wood spacers were used for the outer and inner gypsum panels. A medium-density board was used in combination with the exterior gypsum board. The main idea was that two different materials were used with an air gap in between, so what passed in one could not pass in both. The sensors and camera were then placed inside the box [63].

### 2.2. Data-Gathering Setup

**Figure 2:**
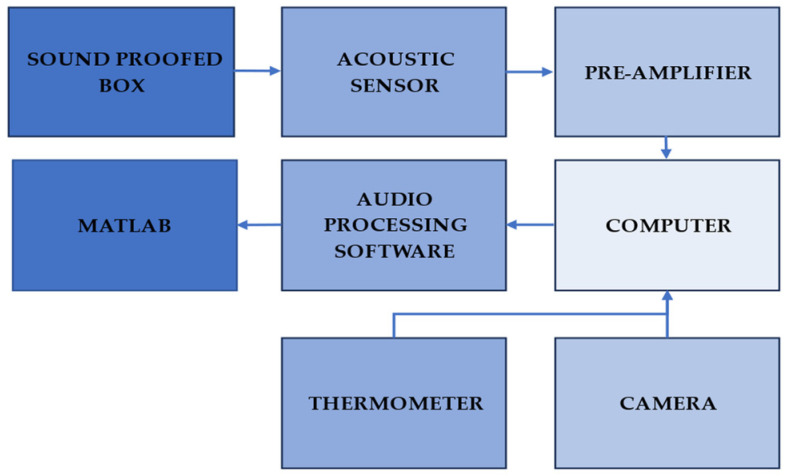
Block diagram for the recording process.

The data-gathering process is shown in Figure 2. The data acquisition started by placing the specimen in the soundproofed box. The box was equipped with an acoustic sensor, thermometer, and camera. The acquired signal was amplified and then recorded on the computer. This recorded signal was then processed using audio software. This audio sample would then be segmented and classified according to the activity of the MPW, as seen in the video recording. The classified audio samples were subjected to the machine learning algorithm developed in MATLAB.

The temperature inside and outside the box were monitored using a wireless thermometer. Monitoring was performed since weevils cannot withstand temperatures exceeding 30 °C. Data gathering was performed at night since this was when the weevil was most active [26,27].

### 2.3. Data-Extraction and Training

**Figure 3:**
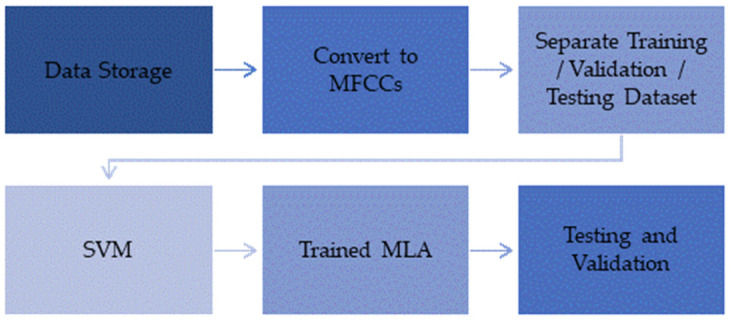
Data extraction and training process.

To be able to extract usable data, classify it, and respond according to the information, machine learning processes were applied. Most machine learning processes utilize a feature extraction method and an algorithm for decision-making. Segmented signals were pre-processed using fast Fourier-transform (FFT) analysis, and features were extracted using Mel-frequency cepstral coefficients (MFCC) and subjected to support vector machines (SVM) for training and validation. The process is shown in Figure 3.

#### 2.3.1. Feature Extraction

**Figure 4:**
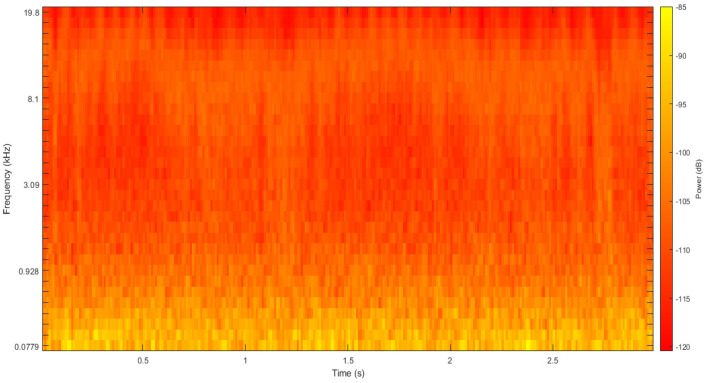
Sample Mel spectrogram of adult MPW walking.

The Mel-frequency cepstrum (MFC) depicts the short-term power spectrum of a signal. The MFC is established on a linear cosine transform of the logarithm power spectrum on a nonlinear Mel scale of frequency. The Mel-frequency cepstral coefficients (MFCCs) make up the MFC. They are derived from a category of cepstral depiction of an audio clip. The main distinction between MFC and Cepstrum is that in MFC, the band of frequencies is uniformly spaced on the Mel scale [67]. In MFCC, the short-term spectral features are obtained by segmenting the audio signals and windowing them in short frames of N samples. Using the fast Fourier transform (FFT), the magnitude spectrum was computed and converted into a group of Mel-scale filter bank outputs. Then, to obtain the MFCCs, a logarithm was used on these bank outputs, followed by a discrete cosine transform [68]. A sample of the processed signal is shown in Figure 4 below.

#### 2.3.2. Machine Learning Algorithm (MLA)

Support vector machine (SVM) was developed to reduce data complexity. Support vector machines (SVM) are efficient and can handle complex data with a constraint function for error minimization, and can still be precise in generalization. They can give reliable classification of unseen data even with minimal training data. Classifiers based on SVM are easy to implement, require minimal parameter tuning, and are unique because of convex optimization and quadratic cost function [69,70,71]. For the data set acquired, 70% was allocated for training and 30% for testing. SVM was implemented in MATLAB.

## 3. Comparison of Available Sensors

Since Palawan is a secluded island, buying from overseas will take a long time, and some shops do not deliver in the Philippines. The study utilized locally available sensors for acoustic characterization of the MPW. This section describes the selection of which available sensors were used to acquire frequencies to describe MPW activity.

Three sensors were identified for the acoustic emission detection of the MPW. These were the Piezoelectric sensor, MEMS, and electret microphone [72]. The sensors were tested in the setup previously described. Each observation lasted for an hour during the night.

**Figure 5:**
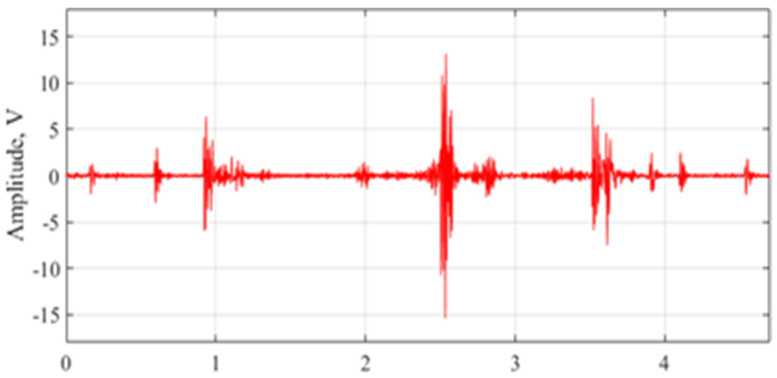
Adult MPW segmented signal [72].

The movement of MPW inside the box was monitored and recorded continuously. Acoustic signals of MPW activity in 5 s segmented samples were gathered and analyzed. A sample waveform of the captured signal from the MEMS microphone is shown in Figure 5.

Segmented signals were pre-processed using fast Fourier-transform (FFT) analysis. The sample signals were normalized to RMS values to allow comparison of the sensor sensitivity. Signal statistics were also computed and compared in Table 1. These values evaluate the performance of the sensors in terms of sensitivity, tolerance, detection of signal covered by noise, and finding fundamental frequencies [73].

**Figure 6:**
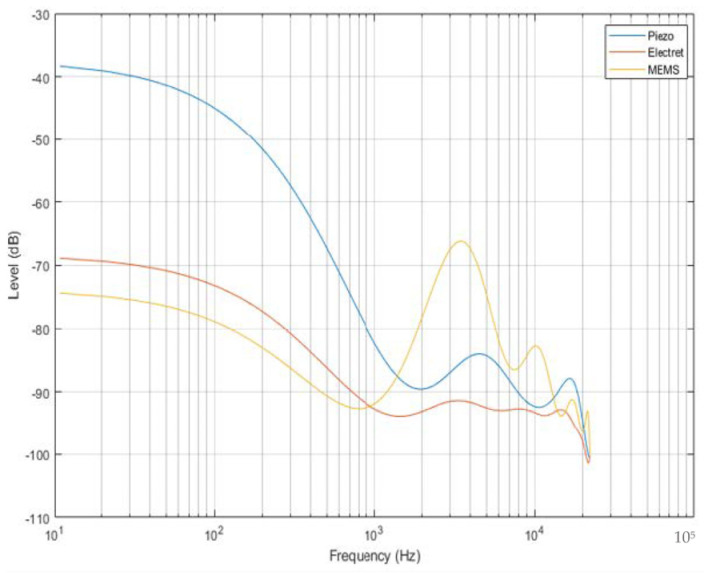
Gain vs. input frequency of the sensors [72].

Figure 6 shows the comparison of the three sensors in a segmented signal of similar activity and duration. It can be observed that there is no significant difference in the sensitivity of the electret and MEMS microphone over lower frequencies. While Piezo is most sensitive to external vibrations, the MEMS microphone has good results over higher frequency values with lesser values of low-frequency noises [72]. The sensitivity dropped at higher frequencies due to the range limitations of the sensors.

During the tests, it was also observed that the Piezo was very sensitive to movement only when the insect was atop the sensor, while the electret microphone captured more noise than insect activity even when the insect was already walking on the sensor itself and the MEMS microphone would register tiny specks when the weevil was walking on it, and slightly larger signal amplitude when it was flying [72]. This study concentrated on using a MEMS sensor to evaluate the acoustic emission of the mango pulp weevil.

## 4. Results

### 4.1. Pupal Activity

The recorded audio and video files were manually inspected and classified according to the demonstrated activity of the specimen inside the soundproof box. The classified audio files were pre-processed using FFT. MPW pupal activity signals show high amplitude frequencies ranging from 1 to 4 kHz.

**Figure 7:**
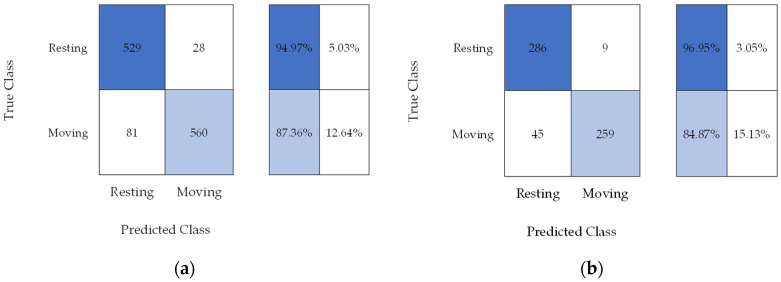
Confusion matrix for SVM classifier in pupal activity (**a**) validation and (**b**) testing.

Classified data sets were validated by an entomologist. These were subjected to the SVM classifier for training, validation, and testing. The confusion matrix is shown in Figure 7. The SVM classifier for pupal activity had an overall accuracy of 90.93%.

Table 2 shows the accuracy of the classifier model for pupal activity. These results indicate that SVM can classify pupal activity in a controlled environment.

### 4.2. Adult Activity

**Figure 8:**
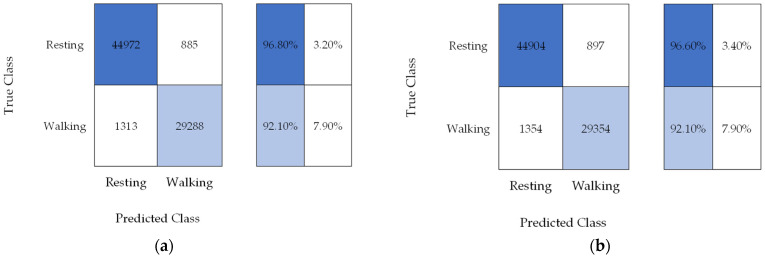
Confusion matrix for SVM in adult activity (**a**) validation and (**b**) testing.

As with the pupa, the recorded signals were also manually inspected and classified according to the MPW activity and validated by the entomologist before pre-processing and machine learning. Figure 8 presents the confusion matrix for adult MPW activity. MPW adult activity signals show high amplitude frequencies ranging from 4 to 5 kHz.

Table 3 shows the accuracy of the classifier model for adult activity, specifically walking. These results indicate that SVM can classify adult MPW activity in a controlled environment.

### 4.3. Adult Mating Activity

**Figure 9:**
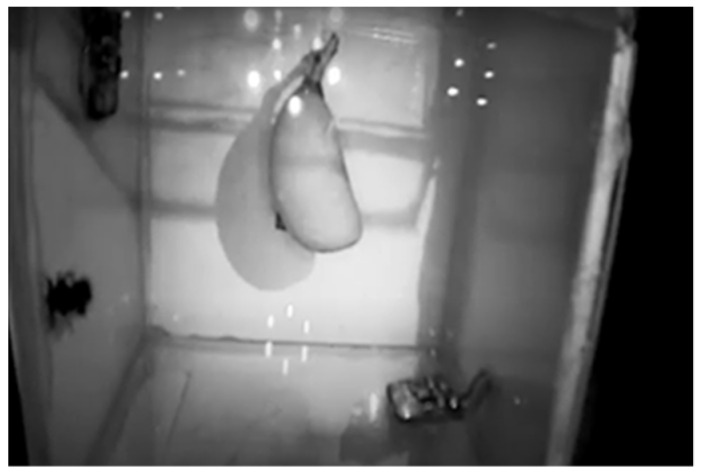
MPW mating stage [28].

The mating activity was classified and segmented using image processing. It is known that the mango pulp weevil mates for 15–20 min at a time [74]. The mating stage starts with the male chasing the female specimen and mounting on its back. As seen in Figure 9, the lower left side of the footage shows two weevil specimens, one male and one female, mounted on top of one another with the mango positioned in the background. All subsequent weevil pairs were observed similarly.

**Figure 10:**
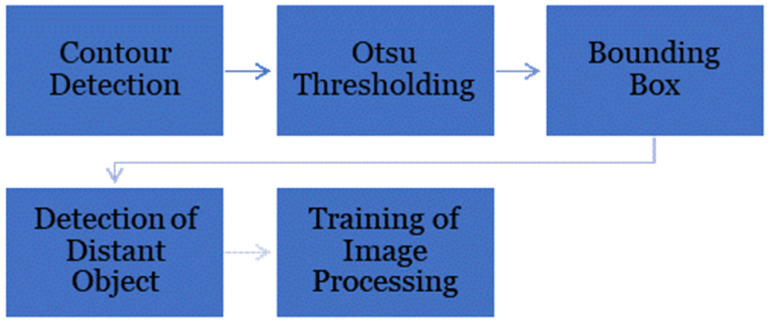
MPW Mating Data Segmentation.

The process of MPW mating segmentation is shown in Figure 10. Classification begins once the video footage is loaded. Simple contour detection was performed to detect the presence of the weevils. Otsu thresholding was then utilized to distinguish the location of the weevils. The region of interest was then reduced by performing a window filter for the area of the resulting mask. Afterward, a bounding box was created to be used as a marker for the location of the weevil throughout the video footage. These markers serve as frames to detect distant objects that could be a source of error. The detection was performed by calculating the distance from the previous frame to the current frame since the weevils cannot move from one place to another with a higher gap within the 1-frame difference. Afterward, image processing using a Kalman filter was trained to automatically segment the video to classify the start of each mating activity. The indicator of the mating activity is when the male and female weevils overlap each other.

**Figure 11:**
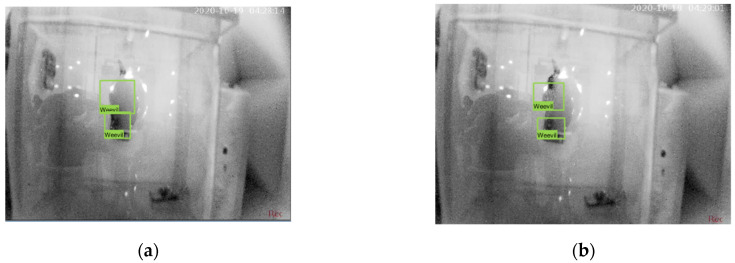
Image processing determining (**a**) mating and (**b**) post-mating [28].

Figure 11a shows the moment when the image processing detects the overlapping of weevils, in which the program also records the timestamp as the start of the mating stage. Figure 11b shows how the program detects the separation of the weevils. The program automatically notes the total duration of the mating stage once the weevils separate.

**Figure 12:**
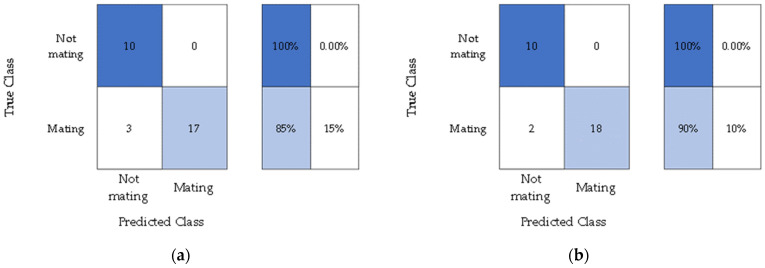
Confusion matrix for SVM in mating activity (**a**) validation and (**b**) testing.

The frequency acquired during the mating stage was 800 Hz to 950 Hz. Figure 12 shows the confusion matrix of the MPW mating activity. In the testing period for the pairs of weevils, some pairs did not mate while on observation. As a result, there were only a few mating instances where 20 were used for the validation and another 20 for testing.

Table 4 shows the accuracy of the classifier model for mating activity. These results indicate that SVM can classify the MPW mating activity in a controlled environment.

## 5. Discussion

A MEMS acoustic sensor was used for the frequency characterization of MPW pupa and adult activity.

MPW pupal activity signals show high amplitude frequencies ranging from 1 kHz to 4 kHz. The frequencies have similarities with the acoustic event of the palm weevil *Rhynchophorus ferrugineus*, with sound impulses having peak frequencies between 1 and 3.8 kHz [75,76]. Pupa stages of the MPW are mostly resting [26,27,29]. But, as observed in the tests, the pupa was somewhat moving or throbbing when the mango was disturbed. This movement is what is detected by the acoustic sensor.

MPW adult activity signals show high amplitude frequencies in the 4 kHz to 5 kHz range. Mango adult weevils are crawlers [26,42]. Upon their development, they stay feeding inside the mango and wait until it falls to the ground for them to crawl back to the tree [26,27]. Then, they will remain in the bark and wait for the next fruiting season.

The frequency acquired during the mating stage is 800 Hz to 950 Hz. Mating mostly happens during the mango fruit development [41,77]. Since weevils do not use chemical cues, these pests must use another means of communication for mating. Mating starts when the male chases the female for a period of time before mounting on the top of it [41].

A machine learning algorithm has been implemented for the classification of weevil activity. SVM was selected for accuracy, even in a small amount of training data [70]. For the MPW pupa, 1198 data samples were used as validation datasets. Out of the 1198 data samples, 641 are moving. Testing was performed in the remaining 599 data samples, where 304 are moving. The overall accuracy of the SVM classifier for pupa activity was 90.93%. For the MPW adult activity, the observation was divided into walking and resting. A total of 76,458 data samples were used for validation, and 76,509 were used for testing. Out of the 152,967 samples, 61,309 were walking data samples. The overall accuracy of the SVM classifier for adult activity was 95.65%.

For the MPW mating activity, the whole file, both audio and video, was fed to the classifier and then processed using the MLA. The main challenge is that the mating process of the weevils had different durations, and some pairs did not mate during the observation. Samples were collected from the multiple occurrences of mating for the weevil pairs. For the total mating occurrences in all subjected weevil pairs, 20 data samples were used for validation and 20 for testing. The overall accuracy of the SVM classifier for the MPW mating activity was 87.50%.

## 6. Conclusions

Most of the studies were on the behavior and life cycle of the MPW and pest-management practices for the said pest. This study has given new insight into applying recent technologies on MPW detection, characterization of frequencies for different activities, and development of a process using a machine learning algorithm for classification.

Acoustic sensing has characterized the mango pulp weevil activity and has given further insights into the behavior of the MPW. Adult MPW frequency registers at 4–5 kHz when walking and 800 to 950 Hz when mating. Machine learning has also been trained to identify the signals for the MPW activity. The trained SVM classifier had an overall accuracy of 89.81%. These results showed that MPW could be acoustically detected.

Further investigation will include detecting the presence of MPW inside the fruit using the MEMS sensor in a laboratory setup to verify the characterization results of this study, as well as utilizing a different type of algorithm, such as autoencoders, for a more accurate result.

## Figures and Tables

**Table 1:** Sensor-recorded audio signal statistics comparison [72].

Factors	Electret	MEMS	Piezo
Minimum value	65.1305	13.5355	32.597
Maximum value	−65.2097	−15.8223	−35.3336
Mean value	0.00046877	0.000047791	−0.00068479
Dynamic range	76.3790 dB	85.5970 dB	85.4947 dB
Crest factor	36.2862 dB	23.9854 dB	30.9638 dB
Autocorrelation	2.9924 s	2.6002 s	2.0403 s

**Table 2:** SVM classifier model accuracy and error percentage for MPW pupal activity.

	Validation Phase	Testing Phase
Accuracy	90.90%	90.98%
Error	9.10%	9.02%

**Table 3:** SVM classifier model accuracy and error percentages for MPW adult activity.

	Validation Phase	Testing Phase
Accuracy	97.13%	97.06%
Error	2.87%	2.94%

**Table 4:** SVM classifier model accuracy and error percentages for MPW Mating.

	Validation Phase	Testing Phase
Accuracy	85%	90%
Error	15%	10%

## Data Availability

Data are available upon request due to restrictions. The data presented in this study are available upon request from the corresponding author.
